# How mindfulness shapes AI competence: a structural equation modeling analysis of mindfulness, AI literacy and behavioral intention in Chinese media students

**DOI:** 10.3389/fpsyg.2025.1652934

**Published:** 2025-08-18

**Authors:** Yanling Lan, Sihang Liu, Linjie Xia

**Affiliations:** School of Film Television and Communication, Xiamen University of Technology, Xiamen, China

**Keywords:** mindfulness, AI literacy, AI behavioral intention (AIBI), media students, media education

## Abstract

**Introduction:**

Artificial Intelligence (AI) literacy, defined as the knowledge and ability to recognize, apply, and evaluate AI, is a key driving force of digital transformation and technological innovation. In the media industry, the demand for “intelligent+” interdisciplinary talent has prompted universities to embed AI literacy training into talent development programs. While curriculum systems have been progressively refined, the challenge remains on how to activate students’ intention to embrace and effectively utilize AI. Mindfulness, a metacognitive trait that enhances cognitive flexibility, self-regulation, and creativity, may contribute to the development of AI literacy, although its specific impact in this progress remains largely unexplored.

**Methods:**

This study constructs the integrated model of “Mindfulness-AI Literacy-Technology Application“. Survey data were collected from 588 media students in China and analyzed using SPSS and SmartPLS to conduct structural equation modeling. AI literacy is comprised of four dimensions: acknowledgment of AI (AAI), AI ethics (AIE), AI collaboration (AIC), and AI self-efficacy (AIS).

**Results:**

Mindfulness significantly and positively influenced AAI, AIE, and AIC, but showed no significant relationship with AIS. It also had a significant direct positive effect on AIBI. Furthermore, AAI and AIC partially mediated the relationship between mindfulness and AIBI.

**Discussion:**

Results confirm that mindfulness is an effective internal pathway for strengthening key AI literacy dimensions and enhancing media students’ intention to apply AI technologies. Incorporating mindfulness interventions into higher media education, aligned with curriculum and practice, could provide a strategic approach to cultivating AI-ready graduates.

## 1 Introduction

Technologies such as artificial intelligence (AI), big data, cloud computing, and large language models have become new productivity, driving the sustainable and high-quality development of various contemporary industries (Lu et al., 2018). In the media industry, the most technologically advanced sector, innovation and upgrades in creation production, content formats, and scene applications have been transformed by AI, the Internet of Things (IoT), and information technology (IT) (Wang et al., 2024). The “intelligent +” media industry pattern has been emerging. Continuously focusing on and participating in technological upgrades and iterations, considering and evaluating the bidirectional effects of technology, and taking action that can enable intelligent “symbiotes” to collaborate in media production, are essential opportunities and challenges for every media practitioner (Hurlburt and Reisman, 2024).

In the process of integrating AI with media, embedding the cultivation of AI literacy and the application of AI technologies into the cultivating framework for media professionals has become an inevitable step in reforming media education. AI is transforming the production of media by enabling the creation of content in multiple formats, including text, visuals, audio, video, and animation. It is also fundamentally redefining the concept of creativity in the media industry (Relmasira et al., 2023). Furthermore, significant progress in AI, particularly in machine-learning technologies, has greatly increased the efficiency and significance of innovation across the media industry (Opdahl et al., 2023). These changes have made the development of AI literacy among media professionals an unprecedented strategic priority. As members of the media workforce in the future, media students must also confront the opportunities and challenges posed by AI transformation. They must build critical thinking and digital technology skills and learn to collaborate effectively with intelligent technologies based on a deep understanding (Vodă et al., 2022). Improving their AI literacy to enhance their professional competitiveness is an urgency for both the growth of the media students and the future of the industry and the growth of the media future.

However, in the context of current media education in universities, although AI literacy courses and AI technology application training are offered, the systematic understanding of internal mechanisms and effective educational outcomes is still lacking. Currently, media education in universities generally focuses on external driving factors such as the curriculum, training and practical experience (Yu, 2024). While the external enabling mechanisms of the curriculum system have gradually improved, there is a demand to establish internal driving mechanisms that activate learners’ intrinsic recognition of, and willingness to adopt AI technologies. Very few studies and practices focus on students’ internal motivational factors (Yim and Su, 2025). Yang et al. (2025) note that the current media education system highlights AI literacy as a vital component in technological applications, innovation work, and media support, yet it disregards the students’ psychological factors and endogenous variables for making impact on AI literacy (Kim et al., 2025). Fan and Zhang (2024) also emphasize that students’ attitudes, enjoyment, cognition and emotional characteristics provide new perspectives for cultivating AI literacy, stressing the importance of focusing on students’ personal interests and feedback.

In order to cultivate smart media talent, it is important to consider both external environmental influencing factors and internal individual driving factors. In the study of factors affecting individual AI literacy, the existence of “knowledge silos” has been found to have a significant impact on students’ ability to learn, share and communicate about AI technologies. In other words, students have concerns about the safety, fairness, responsibility and ethical issues related to the use of these technologies due to the increased uncertainty and risks brought about by the dynamic, autonomous and opaque nature of AI systems’ decision-making processes. The lack of knowledge and knowledge gaps can lead to negative attitudes and difficulties in using AI (Dodds et al., 2025). Studies also suggest that media students may be reluctant to incorporate AI into their courses due to their tendency to avoid potential risks and technological anxiety (Zhu et al., 2025).

Positive mindfulness is regarded as a conscious mental state that has been shown to influence individuals’ ability to make informed decisions. Mindfulness has been found to foster individuals’ ability to adopt positive cognitive appraisals and regulate emotions effectively when facing challenging situations (Liu et al., 2023). Moreover, mindfulness significantly enhances psychological resources such as clarity, resilience, and hope, contributing to greater emotional stability and adaptive functioning (Chiou et al., 2022). In the field of technological applications, positive mindfulness can play a key role in reducing technological stress, emotional exhaustion and intellectual panic, while also enhancing well-being during using technology (Xu et al., 2022; Azpíroz-Dorronsoro et al., 2024). Furthermore, an increasing number of studies have demonstrated that individuals with a positive outlook are highly motivated to innovate. This can enhance the development of learners’ digital and information literacy, as well as their personal well-being and self-confidence in the digital workplace. The development and popularity of digital and information technology have made “Positive Thinking in IT” to be a hot research area. People with positive thinking in IT may focus more on current IT characteristics and explore technical details, applications, and characteristics (Thatcher et al., 2018). At the same time, in the dynamic and evolving field of communication studies, the integration of mindfulness has emerged as a transformative influence, shaping interpersonal, group, mediated, and cross-cultural communication. Mindfulness has been shown to significantly enhance individuals’ attentional control and empathetic understanding, thereby facilitating more effective adaptation to diverse and complex communication environments (Chen et al., 2024).

This study will examine the relationship between mindfulness as an individual psychological trait and AI literacy as a cognition and application of intelligence technologies. It will use students’ levels of mindfulness as an indicator of their psychological willingness, exploring how media students with different levels of mindfulness exhibit varying performances in terms of AI literacy and their intention to use AI tools. Taking a dual perspective that considers both the students’ internal characteristics and external environmental influences, the study will investigate ways to promote students’ development in AI literacy, equipping students to meet the demands of the smart media era. Adopting a micro-level, individual perspective, the study aims to shed new light on providing theoretical support for developing AI literacy and cultivating intelligent media professionals.

## 2 Theoretical review and research hypothesis

### 2.1 Mindfulness

Mindfulness is commonly defined as “non-judgmental awareness of present experiences.” The term was first introduced in 1881 by the British scholar Rhys Davids, who discovered and translated the Buddhist concept of “Sati,” meaning “memory, recollection, or awareness of certain facts” (Sun, 2014). Initially, mindfulness emphasized its experiential nature. Over time, however, it has become specifically associated with the generation and effects of mindfulness. It now primarily refers to the heightened awareness and attention that individuals develop during and after mindfulness practice with regard to their current events and experiences (Lama, 2005). Subsequent research has further demonstrated that mindfulness significantly influences individual qualities such as creativity, flow, and emotional regulation. This impact is rooted in the requirement for individuals to maintain open and receptive awareness of sensory and external stimuli during mindfulness practice, thereby promoting cognitive development and enhancing personal qualities (Chen H. et al., 2022). Research by Pérez-Peña et al. (2022) suggests that mindfulness can improve an individual’s awareness of their own body. Furthermore, mindfulness plays a significant moderating role in cultivating personal qualities such as experience avoidance, self-reflection, self-efficacy and self-differentiation. This demonstrates a positive and significant correlation between mindfulness and cognitive and literacy development.

Moreover, experimental applications of mindfulness training to enhance personal competency and literacy have been effectively implemented across various sectors. Their study also found a significant, positive correlation between mindfulness and greater psychological resilience, lower perceived stress, and the adoption of more effective coping strategies. Salvado et al. (2021) applied mindfulness-based interventions within the healthcare sector to alleviate burnout among professionals and improve both their professional competence and personal qualities. Janssen et al. (2020) assessed the feasibility and acceptability of mindfulness in medical settings, finding that mindfulness significantly reduces personal stress levels, negative emotions, and enhances self-efficacy. In the context of higher education, mindfulness has been recognized as a critical variable in fostering character development. Guidetti et al. (2019) conceptualized mindfulness as an internal resource that interacts with both learning demands and cognitive capacities. By alleviating the academic burden and reshaping cognitive meaning, mindfulness contributes to enhancing students’ psychological resilience. Furthermore, Li (2025) integrated mindfulness into higher education curriculum design and pedagogical norms, demonstrating its positive effects on students’ self-confidence, responsibility, collaboration, and creativity, particularly in improving focus and empathy.

### 2.2 AI literacy

Artificial Intelligence (AI) literacy is an interdisciplinary competency that spans multiple fields, including computer science, psychology, sociology, philosophy, and others. In the context of the widespread integration and popularization of intelligent technologies, AI literacy has become a crucial foundation for both individual and societal development (Wang et al., 2023). Ng et al. (2021) established the theoretical framework for defining AI literacy, identifying four core components: knowledge and understanding of AI, practical application of AI, critical evaluation of AI, and ethical engagement with AI. Building on this, Laupichler et al. (2023) conceptualized AI literacy into three key dimensions: cognitive understanding of AI, critical regulation of AI, and collaboration and application of AI. The relative studies collectively emphasize a conceptualization of AI literacy that primarily focuses on cognitive understanding and knowledge of AI. Additionally, the evaluation and regulation of AI are considered unique and essential elements. The collaborative use of AI is regarded as a central part of advanced AI literacy, while AI efficacy has also been identified as an important factor by subsequent researchers. Notably, this framework has been translated into both English and Spanish versions, making it one of the most widely applied measurement systems in the field (Lintner, 2024).

Dai et al. (2020) emphasized that AI literacy can significantly enhance students’ self-confidence and their understanding of the relevance of technology, while simultaneously reducing anxiety and fear associated with technology. Ultimately, this has a positive impact on their intention to use AI tools. More recently, Pan et al. (2025) integrated AI literacy into the Technology Acceptance Model (TAM), demonstrating its positive influence on factors such as perceived usefulness, perceived ease of use, and behavioral intentions. This enables individuals to assess the reliability, effectiveness, and practicality of AI tools, thereby ensuring fairness, convenience, and rationality in their usage (Tenberga and Daniela, 2024). Furthermore, AI literacy serves as a bridge between multidisciplinary fields and diverse participants within the AI domain, emerging as a key trend shaping the future of education, industry intelligence, and digital transformation (Cabero-Almenara et al., 2025; Newman-Griffis, 2025).

In the era of artificial intelligence, the new media creates and disseminates content in more efficient and diverse ways. This contributes not only to the transformation of the media industry but also to the evolution of media education. On the one hand, artificial intelligence (AI) enhances media productivity and content quality, increases audience engagement, and generates revenue streams, thereby fostering economic growth within the media industry. On the other hand, AI may also undermine media norms and practices by diminishing the accuracy, authenticity, neutrality, and transparency of information. This could, in turn, erode public trust in the media and even weaken social cohesion (Aitamurto and Boyles, 2025). Enhancing the AI literacy of media professionals is essential for improving their ability to verify information and control the dissemination of content (Harambam et al., 2018). As with any revolutionary new technology, generative AI presents both exciting opportunities and challenging dilemmas. Therefore, cultivating and enhancing the AI literacy of media professionals is crucial and urgent (Quinonez and Meij, 2024).

### 2.3 The impact of mindfulness on AI literacy and application

Mindfulness significantly impacts individual literacy and behavioral capabilities, and is equally effective in developing AI literacy and using intelligent technologies (Chen et al., 2025). An increasing number of research has demonstrated that mindfulness training significantly influences the development of literacy related to intelligent and digital technologies, as well as enhancing memorial and cognitive abilities (Liebherr et al., 2024). Kurki et al. (2021) have shown that mindfulness can reduce stress, improve AI literacy, and enhance well-being among students, thereby facilitating the integration of digital transformation plans into higher education. Wang (2025) found that digital mindfulness can reduce the cognitive load associated with technology for students, enhance their cognitive awareness, and promote their understanding of the complexity of technological tasks. Rodrigo et al. (2024) argue that mindfulness contributes to cultivating digital resilience and AI literacy. Individuals with higher levels of mindfulness tend to demonstrate a clearer understanding of the present moment. They are less likely to exhibit impulsive behavior and are less prone to anxiety and emotional distress induced by emerging technologies. This, in turn, facilitates the development of their AI literacy (Tan et al., 2022). On the other hand, mindfulness provides individuals with the opportunity to overcome the “dark side” of technology and significantly reduces technological stress. Conversely, individuals with lower levels of mindfulness tend to exhibit less resilience when facing technological stress (Rohwer et al., 2022). In environments of work, mindfulness can reduce digital stress, improve employee well-being, and promote the acceptance of technology (Taylor and Millear, 2016). Pflügner and Maier (2019) conducted a detailed study on the relationship between mindfulness and technology usage. They revealed that employees with higher levels of mindfulness exhibited lower levels of techno-stress, including feelings of overload, complexity, invasiveness and uncertainty.

Existing research has shown that mindfulness, when integrated into AI technologies and their application, can alleviate the impact of technological stressors. Consequently, mindfulness could play a significant role in facilitating the adoption of intelligent technologies and fostering intelligent work environments (Thatcher et al., 2018). Atoy et al. (2020) examined the relationship between digital literacy, online information-searching strategies, and mindfulness among Philippine university students. Building on existing research regarding prior knowledge, the availability of information and communication technology resources, and the validation of intelligent technologies, they identified new endogenous variables. They incorporated mindfulness into the relationship between AI literacy and ICT (‌Information and Communications Technology)‌, highlighting the positive correlation between mindfulness and both AI literacy and technology usage. Nascimento et al. (2024) identified the antecedents and outcomes of techno-stress in higher education. They specifically noted that individuals’ mindfulness positively impacts the perceived usefulness and reliability of technologies, contributing to the development of AI literacy and reducing technological stress. Koch and Fehlmann (2025) examined the factors affecting the digital performance of university staff in various media tasks. They found that, when given the necessary time and resources, employees with higher levels of mindfulness exhibited higher AI literacy and could utilize digital information resources more effectively.

### 2.4 Research questions and hypotheses

Based on the existing research, it is clear that mindfulness plays a significant role in cultivating AI literacy and the willingness to use AI tools, serving as a psychological foundation for enhancing individual AI literacy. However, several gaps exist in current studies on how to enhance AI literacy and willingness to apply AI among media students. These gaps can be summarized as follows: (1) Research Perspective: A substantial portion of existing studies primarily focuses on external environmental factors, such as the analysis of higher education curricula, teaching methods, and the impact of technological changes in the AI era on individuals. However, these studies often overlook the potential influence of intrinsic personal drivers, creativity, and awareness from an endogenous perspective. (2) Research Subject: Many studies focus on professionals from various industries, such as healthcare, digital work, and media. However, there is a noticeable lack of research focusing on higher education students, particularly those in fields directly impacted by industry changes. This group represents the “reservoir” of future workers in the labor market, specifically media students who will be profoundly affected by these changes. (3) Research Content: There is a lack of systematic analysis grounded in established theoretical frameworks. Furthermore, there are considerable opportunities to refine and explore theoretical models and empirical data in this area. Therefore, this study aims to investigate the following research questions:(1) Does mindfulness have a significant impact on AI literacy?(2) Can mindfulness directly influence the intention to use AI tools?(3) How does AI literacy affect the intention to use AI tools?

**Figure 1:**
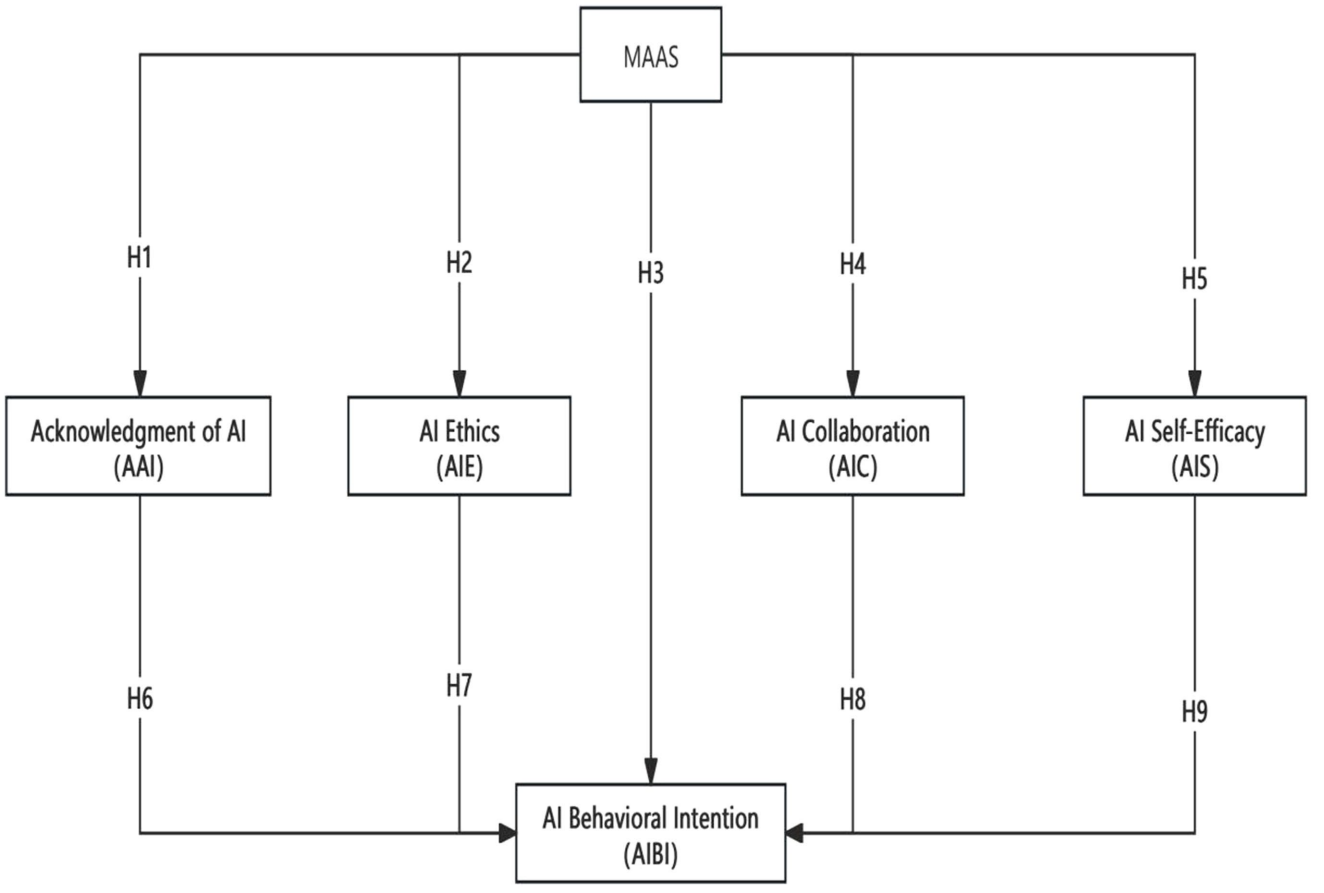
Integrated model of “Mindfulness—AI literacy—technology adoption intention”.

Based on the above literature review, this study has developed a theoretical framework (Figure 1) and proposed the related research hypotheses.

*H1*: Mindfulness has a significant positive effect on individuals’ acknowledgement of Artificial Intelligence.

*H2*: Mindfulness has a significant positive effect on individuals’ evaluation of the ethics related to Artificial Intelligence.

*H3*: Mindfulness has a significant positive effect on individuals’ behavioral intention to use AI tools.

*H4*: Mindfulness has a significant positive effect on individuals’ engagement in AI collaboration.

*H5*: Mindfulness has a significant positive effect on individuals’ perceived self-efficacy of Human-AI interactions.

*H6*: Acknowledgement of AI has a significant positive effect on individuals’ behavioral intention to use AI tools, and mediates the relationship between mindfulness and the intention to use AI tools.

*H7*: Evaluation of the ethics related to AI has a significant positive effect on individuals’ behavioral intention to use AI tools, and mediates the relationship between mindfulness and the intention to use AI tools.

*H8*: Engagement in AI collaboration has a significant positive effect on individuals’ behavioral intention to use AI tools, and mediates the relationship between mindfulness and the intention to use AI tools.

*H9*: Perceived self-efficacy in Artificial Intelligence of Human-AI interactions has a significant positive effect on individuals’ behavioral intention to use AI tools, and mediates the relationship between mindfulness and the intention to use AI tools.

## 3 Research subjects and methodology

### 3.1 Research subjects

The participants in this study were Chinese media students, including students from the majors in journalism and communication, television and film, and digital media art at both the undergraduate and graduate levels. Advances in artificial intelligence technologies have driven fundamental, even disruptive, changes in the media industry (Nguyen and Hekman, 2022). When media students use artificial intelligence technology, they must be highly dependent on their own perceptions and assessments of the usefulness, controllability, and value of the technology to ensure the ethical integrity and content accuracy of the generated material (Newman, 2022). Therefore, compared with students of other majors, media students face higher psychological complexity and situational sensitivity when using AI tools.

The minimum sample size for this study was determined using the G*Power tool (Faul et al., 2009). With parameters set to a moderate effect size of 0.15, an error rate of 0.05, an effect strength of 0.8, and five predictors, the minimum sample size required for credible results was 92. The sample was selected using stratified random sampling, with strata based primarily on participants’ major and academic year, ensuring a representative distribution of students across the study’s relevant subgroups.

This study utilized the Questionnaire Star platform to design and distribute the questionnaire in March 2025. The distribution was conducted offline, with face-to-face administration, on-site completion, and collection. Of the 610 returned questionnaires, invalid responses were identified and excluded based on the following criteria: (1) key items were missing or unanswered, and (2) the response time was significantly shorter than the average time (i.e., less than 1 min). After excluding 21 invalid responses, 589 valid questionnaires remained, yielding a response rate of 96.7%. For data analysis, SmartPLS (V4.1) was used to assess the direct and indirect effects among variables in the structural equation model.

### 3.2 Research design

The questionnaire used in this study consists of three sections: (1) demographic information of the respondents, (2) Mindfulness (MAAS), and (3) key variables related to AI literacy, including Acknowledgement of AI (AAI), Ethics of AI (AIE), Collaboration in AI (AIC), and Self-Efficacy of AI(AIS). To ensure the accuracy and objectivity of the questionnaire translation and design, the items were adapted from relevant literature and translated with great care. Prior to distribution, a rigorous back-translation process was employed, involving one PhD student and two graduate students who reviewed the translations to ensure both linguistic and conceptual accuracy. All independent variables were measured using a 5-point Likert scale (1 = Strongly Disagree, 2 = Disagree, 3 = Neutral, 4 = Agree, 5 = Strongly Agree).

Mindfulness Scale: The Mindful Attention Awareness Scale (MAAS), developed by Brown and Ryan (2003) was used to assess individuals’ mindfulness characteristics. The scale consists of 15 items, each rated on a 6-point scale. Among the various tools used to assess mindfulness traits, the MAAS is one of the most widely adopted in research. To minimize potential bias and maintain objectivity, this study used a 5-point Likert scale (1 = Strongly Disagree, 5 = Strongly Agree), with lower scores indicating greater mindfulness. In this study, Cronbach’s *α* for the mindfulness scale was 0.921 (see Table 1).

**Table 1:** Analysis of reliability and validity about questionnaire.

Construct	Cronbach’s alpha	KMO
MAAS	0.921	0.935
Acknowledge artificial intelligence	0.908	
Artificial intelligence ethics	0.828	
Artificial intelligence collaboration	0.841	
Artificial intelligence self-efficacy	0.868	
Artificial intelligence behavioral intention	0.85	

Acknowledgement of Artificial Intelligence Scale: The AI Literacy Scale, developed by Kurki et al. (2021), includes the following dimensions: cognitive understanding of AI, application of AI, evaluation of AI applications, and AI ethics. The validity of the scale was verified using KMO and Bartlett’s tests. Zhao et al. (2022) further refined the scale, specifically focusing on the Cognition and Understanding of AI dimension. They used a 5-point Likert scale to measure AI literacy levels, with a Cronbach’s alpha of 0.963, indicating high reliability and validity. In this study, we adapted the Cognition and Understanding of AI scale from Zhao et al. (2022), continuing with a 5-point Likert scale ranging from “Strongly Disagree” to “Strongly Agree.” The scale consists of four items. In this study, Cronbach’s *α* for the Cognition and Understanding of AI Scale was 0.829 (see Table 1).

Ethics of Artificial Intelligence Scale: The Artificial Intelligence Literacy Scale for Chinese College Students (AILS-CCS) was developed and validated by Ma and Chen (2024). The scale underwent expert validation and was refined through focus group interviews to optimize its initial items. The final version of the scale includes four dimensions, one of which is the Evaluation Dimension, which measures the ability to critically assess AI-generated content and evaluate the limitations of AI tools themselves. All factors loading exceeded 0.7, and the AVE values for the scale were all greater than 0.5, indicating strong convergent validity. In this study, Evaluation of AI Scale was adapted, and Cronbach’s *α* for this scale was 0.828 (see Table 1).

Collaboration in Artificial Intelligence Scale: The AI Collaboration Scale was developed by Wang et al. (2023) to measure user competence in AI collaboration. This scale consists of three items and has been validated for reliability and validity through both exploratory and confirmatory factor analysis (EFA & CFA). The development of this scale provides a scientifically sound and practical quantitative tool for assessing overall AI operation and proficiency. In this study, Cronbach’s *α* for the Collaboration in AI Scale was 0.841 (see Table 1).

Self-Efficacy in Artificial Intelligence Scale: The AI Self-Efficacy Scale was initially developed by Compeau and Higgins (1995) as a measure of computer self-efficacy, which assesses individuals’ perceived abilities to use computers effectively. This scale was further validated by Cassidy and Eachus (2002), who extended the concept of computer self-efficacy by exploring the relationship between self-efficacy, gender, and experience with computers. Wang and Chuang (2024) later adapted this foundational work and developed the Artificial Intelligence Self-Efficacy Scale. Drawing on previous research on computer self-efficacy, their scale was validated using confirmatory factor analysis (CFA), demonstrating strong structural validity and internal consistency. In this study, Cronbach’s *α* for the Self-Efficacy in AI Scale was 0.868 (see Table 1).

## 4 Data analysis

The data in this study were analyzed descriptively using SPSS29 software, and the reliability and discriminant validity of the research models were assessed using SmartPLS (V4.1). The validated structural equation models were analyzed against paths, with the significance levels set at *p* < 0.05; *p* < 0.01; *p* < 0.001, and through SmartPLS in the Bootstrapping for mediation effect validation.

### 4.1 Description of demographic variables

The descriptive statistics revealed the following sample demographics (see Table 2): Gender distribution was 22% male (130 participants) and 78% female (458 participants). In terms of grade level, the majority (84%) were undergraduate students (493 participants). The distribution across majors was as follows: Television and Film (241 participants), Digital Media (159 participants), and Journalism and Communication (188 participants).

**Table 2:** Frequency analysis of demographic variables.

Variable	Option	Percentage	Mean	Variance
Gender	Man	22.00%	1.78	0.173
	Women	78.00%		
Qualification	Undergraduate	84.00%	1.16	0.145
	Postgraduate	15.00%		
Major	Drama, film and television	41%	8.90	0.809
	Digital media	27%		
	Journalism and communication	32%		

### 4.2 Model reliability and validity testing

To assess the reliability of the proposed structural equation model, we used Composite Reliability (CR) and Average Variance Extracted (AVE). As shown in Table 3, the CR values for all constructs exceeded the recommended threshold of 0.70. Additionally, the AVE values for most items were above the standard threshold of 0.50. These results indicate that the scale demonstrates adequate convergent validity and internal reliability, thus supporting further path analysis in the structural equation model.

**Table 3:** Reliability and convergence effectiveness of variables.

Reliability	Convergent validity		
Items	Cronbach alpha	CR	AVE
AIBI	0.852	0.858	0.771
AIC	0.838	0.881	0.752
AIE	0.82	0.889	0.728
AIS	0.858	0.913	0.778
MA	0.926	0.935	0.488
AAI	0.908	0.908	0.783

To further validate the convergent validity of the structural equation model, this study used the square root of the AVE values in subsequent analyses. As shown in Table 4, the square root of the AVE for all first-order variables exceeds the correlation coefficients between each variable and the others, further confirming the strong discriminant validity among the factors.

**Table 4:** Fournier lackel standard.

Fournier- lackel values	AIBI	AIC	AIE	AIS	MA	PU
AIBI	0.878					
AIC	0.503	0.867				
AIE	0.417	0.423	0.853			
AIS	0.066	0.038	0.011	0.428		
MA	0.173	0.242	0.11	−0.076	0.698	
AAI	0.731	0.507	0.476	0.035	0.103	0.885

SmartPLS was used to conduct an HTMT analysis to assess the discriminant validity of the scales. The resulting HTMT ratio values (see Table 5) show that all the variables demonstrate good discriminant validity, as all HTMT values are below 0.85. This indicates that the variables are conceptually independent and can independently explain different aspects of the study. Therefore, from a discriminant validity perspective, these variables exhibit both validity and reliability in this research.

**Table 5:** HTMT ratio numerical.

HTMT values	AIBI	AIC	AIE	AIS	MA	PU
AIBI						
AIC	0.616					
AIE	0.493	0.498				
AIS	0.044	0.082	0.06			
MA	0.175	0.226	0.106	0.057		
AAI	0.83	0.603	0.555	0.038	0.103	

### 4.3 Multi-collinearity test

The data collected through the survey may be subject to common method bias, which could arise from the design of the questionnaire items. To mitigate this potential issue, a multicollinearity test, using the variance inflation factor (VIF), was conducted on the six factors extracted for this study. The VIF values for all factors were found to be below the threshold of 3.3. Therefore, the results indicate that the current measurement is not significantly impacted by multicollinearity issues.

### 4.4 Model evaluation and interpretability

Table 6 summarizes the theoretical effect sizes for R2 and f2. The R2 value represents the coefficient of determination for the endogenous variables. An R2 greater than 0.67 indicates strong explanatory power of the model; values between 0.33 and 0.67 suggest moderate explanatory power; and R2 values below 0.19 indicate weak explanatory power. The f2 value reflects the effect size of the exogenous variables on the endogenous variables. An f2 greater than 0.35 represents a large effect size; values between 0.15 and 0.35 indicate a medium effect size; and f2 values less than 0.15 represent a small effect size. Based on the results presented in Table 6, the structural model of this study demonstrates acceptable levels of validity.

**Table 6:** Theoretical effect sizes for R^2^ and F^2^.

R^2^		F^2^					
		AIBI	AIC	AIE	AIS	MA	AAI
AIBI	0.564						
AIC	0.522	0.052					
AIE	0.112	0.042					
AIS	0.115	0.122					
MA			0.055	0.127	0.158		0.124
AAI	0.111	0.543					

### 4.5 Hypothesis testing

Table 7 presents the variances, T-values, and *p*-values (indicating significance) for each path, calculated using the bootstrap method in SmartPLS with 5,000 resamples. The data analysis provides clear evidence that mindfulness has a significant positive effect on cognition and understanding of artificial intelligence (AI). As shown in Figure 1, the research framework reveals that mindfulness has a significant direct impact on AI cognition and understanding, AI collaboration, and the evaluation of AI. Furthermore, mindfulness exerts a significant indirect effect on the practical application of AI. Cognition and understanding of AI, as well as AI collaboration, serve as mediating variables, generating a significant partial mediation effect.

**Table 7:** Path coefficients of the research framework.

β	SD	Confidence interval				Significance
		2.50%	97.50%	t	p	
Direct effects						
MA-AIC	0.043	0.179	0.329	5.325	0	<0.001
MA-AIE	0.048	0.048	0.206	2.263	0.024	<0.05
MA-AIS	0.077	−0.142	0.128	0.894	0.372	>0.05
MA-AAI	0.047	0.007	0.2	2.261	0.024	<0.05
AAI-AIBI	0.028	0.674	0.783	26.355	0	<0.001
AIC-AIBI	0.039	0.107	0.26	4.696	0	<0.001
AIE-AIBI	0.033	−0.019	0.112	1.385	0.166	>0.05
AIS-AIBI	0.036	−0.075	0.063	0.748	0.454	>0.05
Indirect effect						
MA-AAI-AIBI	0.036	0.005	0.15	2.709	0.038	<0.05
MA-AIC-AIBI	0.013	0.022	0.073	3.158	0.002	<0.05
MA-AIS-AIBI	0.003	0.001	0.005	0.688	0.492	>0.05
MA-AIE-AIBI	0.005	0.002	0.016	1.042	0.298	>0.05
Total effect						
MA-AIBI	0.036	0.005	0.15	2.079	0.038	<0.05

### 4.6 Data analysis results

The data analysis yielded the following findings regarding the effects of mindfulness on various aspects of artificial intelligence (AI): Mindfulness and AAI (Acknowledgment of AI): The path coefficient was *p* = 0.024, *p* < 0.05 (SD = 0.047, t = 2.261), indicating a significant positive effect of mindfulness on cognition and understanding of AI. Mindfulness and AIE (Ethics of AI): The path coefficient was *p* = 0.024, *p* < 0.05 (SD = 0.048, t = 2.263), suggesting that mindfulness has a significant positive influence on the evaluation of AI. Mindfulness and AIC (AI Collaboration): The path coefficient was *p* = 0, *p* < 0.001 (SD = 0.043, t = 5.325), indicating a significant positive effect of mindfulness on AI collaboration. Mindfulness and AIS (AI Self-efficacy): The path coefficient was *p* = 0.372, *p* > 0.05 (SD = 0.077, t = 0.894), suggesting that mindfulness does not have a significant effect on AI self-efficacy. Regarding the influence of Acknowledgmen of AI on AIBI (AI Behavioral Intention), the path coefficient was *p* = 0, *p* < 0.001 (SD = 0.028, t = 26.355), indicating a significant positive effect. Additionally, in the MA-AAI-AIBI mediation path, the path coefficient was *p* = 0.038, *p* < 0.05 (SD = 0.036, t = 2.709), suggesting that cognition and understanding of AI serve as a mediator in the relationship between mindfulness and the intention to use AI. For the influence of AI Collaboration on AIBI, the path coefficient was *p* = 0, *p* < 0.001 (SD = 0.039, t = 4.696), showing a significant positive impact on AIBI. In the MA-AIC-AIBI mediation path, the path coefficient was *p* = 0.002, *p* < 0.05 (SD = 0.013, t = 3.158), indicating that AI collaboration also mediates the relationship between mindfulness and the intention to use AI. For the effect of Ethics of AI on AIBI, the path coefficient was *p* = 0.166, *p* > 0.05 (SD = 0.033, t = 1.385), indicating no significant impact. Similarly, in the MA-AIE-AIBI mediation path, the path coefficient was *p* = 0.298, p > 0.05 (SD = 0.005, t = 1.042), suggesting that evaluation does not mediate the relationship between mindfulness and the intention to use AI tools. For the effect of AI self-efficacy on AIBI, the path coefficient was *p* = 0.454, *p* > 0.05 (SD = 0.036, t = 0.748), indicating no significant effect. Furthermore, in the MA-AIS-AIBI mediation path, the path coefficient was *p* = 0.0492, *p* > 0.05 (SD = 0.003, t = 0.688), indicating that AI self-efficacy does not mediate the relationship between mindfulness and the intention to use AI. Finally, in the relationship between Mindfulness and AI Behavioral Intention, the path coefficient was *p* = 0.038, *p* < 0.05 (SD = 0.036, t = 2.079), suggesting a significant positive effect of mindfulness on the intention to use AI tools.

**Figure 2:**
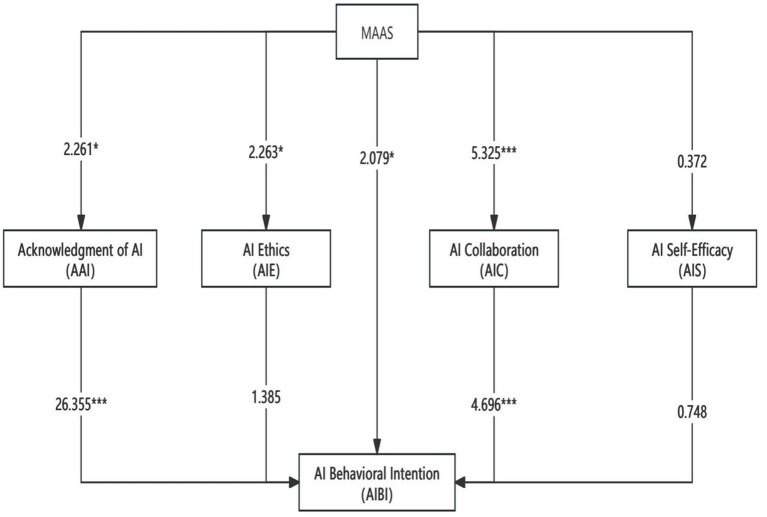
Research framework.

Based on these results, hypotheses H1, H2, H3, H4, H6, and H8 are supported, while hypotheses H5, H7, and H9 are not validated. The final theoretical model of this study is presented in Figure 2.

## 5 Research discussion

### 5.1 Mindfulness and AI literacy

A significant positive correlation was found between mindfulness levels and various aspects of AI literacy, including acknowledgement of AI, AI collaboration, and AI Ethics. These findings support the research hypothesis. Previous studies have shown that higher levels of mindfulness are associated with greater digital literacy and AI acknowledgement, with mindfulness playing a key role in fostering AI literacy (Chen et al., 2025). Thatcher et al. (2018) developed a method for measuring technological mindfulness through an empirical study. Their findings indicate that technological mindfulness significantly enhances the understanding, cognition, and sustained use of new technologies. Liu et al. (2025) employed controlled experiments to examine the relationship between mindfulness and stress and found that digital mindfulness interventions can effectively reduce anxiety and stress across various settings by enhancing individuals’ concentration and emotional regulation. Their findings also suggest that digital mindfulness contributes to improved personal well-being and increased professional productivity. Specifically, mindfulness helps individuals expand their attention span and moment-to-moment awareness, while also facilitating the accumulation of wisdom, experience, and knowledge. This process, in turn, promotes technological cognition and AI literacy (Rodrigo et al., 2024). In addition, mindfulness not only improves individual literacy but also fosters digital resilience. The mindfulness trait enables individuals to focus more deeply on the information they encounter, fostering autonomous awareness of intelligent technologies. This, in turn, contributes to the development of skills necessary for evaluating AI technologies. Within the field of Human-Computer Interaction (HCI), researchers define mindfulness as the mental state of experiencing the present moment. Studies suggest that mindfulness enhances interactions with technology by boosting attention, presence, awareness of bodily sensations, and moment-to-moment consciousness (Terzimehić et al., 2019). In higher education, the growing use of digital and intelligent technologies has led to the creation of virtual learning environments. This evolution, however, introduces the dual challenge of fostering AI literacy and applying intelligent technologies. Mindfulness can enhance students’ understanding of intelligent technologies, improve their AI literacy, and help them cope with stress, thereby promoting overall well-being (Norabuena-Figueroa et al., 2025). Mindfulness is defined as the intentional cultivation of non-judgmental awareness in the present moment. Individuals with higher mindfulness tend to exhibit greater awareness and concentration, allowing them to break free from traditional thought patterns and engage in innovative, objective analysis. As a result, they are more likely to accept new ideas.

However, there is no significant correlation between mindfulness and AI self-efficacy. Digital self-efficacy, which is defined as an individual’s perception of their ability to use digital devices and services, has become increasingly important as AI and algorithms are integrated into all forms of digital technology (Kim, 2025). The effectiveness of AI is influenced by various factors, including environmental conditions, personality traits, and leadership qualities. Since mindfulness is primarily a personality trait, it may not directly impact an individual’s ability to effectively use AI technologies (Lin et al., 2022). Mindfulness primarily involves non-judgmental awareness of the mind, body, and external environment in the present moment. Cultivating mindfulness encompasses three key components: non-judgment, living in the present moment, and awareness of both internal and external stimuli (Liu et al., 2022). Due to its transient and non-critical nature, however, mindfulness is unlikely to have a significant direct impact on the complex and multifaceted construct of AI self-efficacy.

### 5.2 AI literacy and the application of AI technology

This study reveals a significant positive correlation between cognitive ability, understanding of artificial intelligence (AI), AI collaboration, and the application of AI technologies. These findings are consistent with existing theories and research. Cognition refers to the mental processes through which individuals understand, modify, and develop knowledge. Students’ cognitive abilities significantly influence their learning methods, problem-solving strategies, and critical thinking styles. As technology becomes more complex, students with stronger beliefs in their technological abilities tend to engage in more in-depth inquiry, self-regulation, and a better understanding of technology (Hofer and Pintrich, 2002). Zhao et al. (2022) further emphasize the importance of AI understanding in educational contexts. They argue that grasping the fundamental concepts, knowledge, information, and attitudes related to AI is essential for effectively applying AI technologies. Random or unintentional use of AI can diminish students’ willingness to engage with these tools. Therefore, enhancing students’ understanding of smart tools before they use them can increase their willingness to adopt and continue using these technologies. Rapanta et al. (2020) argue that fostering a deeper understanding of AI through reflection can motivate students to incorporate AI tools into their learning processes. However, students’ ability to collaborate with AI tools presents greater challenges than their understanding of AI, making it a critical factor in their acceptance of these technologies (Huang, 2021). Hwang et al. (2023) highlight that students’ perceptions of the usefulness and ease of use of intelligent tools, as well as their capacity to collaborate with AI, are crucial for success in the AI era. High cognitive abilities and effective collaboration with AI tools greatly promote the acceptance and application of these technologies, particularly in higher education.

However, the self-efficacy and evaluation of artificial intelligence (AI) do not significantly impact students’ actual willingness to use AI tools. On the one hand, the specification and evaluation of AI are extremely complex and challenging in practical applications. A user’s evaluation of AI involves not only their reliance on the technology itself but also their perceptions of the developers behind the AI system. Moreover, empirical evidence regarding how characteristics such as interpretability and transparency affect users’ actual behavior remains scarce and unclear. Therefore, the standardization and evaluation of AI must account for broader considerations, such as the development and practical application environments of AI systems. Only through further examination and validation of normative principles, assessment rules, and the potential relationships between them can we fully understand their impact on users’ willingness to engage with smart tools (Chen et al., 2023). On the other hand, the construction of self-efficacy has been recognized as a key factor influencing individuals’ willingness to adopt technology. However, existing studies have not yet demonstrated a significant positive correlation between general self-efficacy and the willingness to use specific smart tools. In particular, few studies have explored the role of self-efficacy in bridging the gap between smart technology and behavioral intentions (Yao and Wang, 2024). McGuire et al. (2024) specifically analyze the relationship between organizational intelligence efficacy and tool application. They point out that the collaborative relationship between “human and artificial intelligence” is multifaceted and complex, making the human-machine relationship a critical factor in the effectiveness of AI. When an individual relies solely on intelligent technology, they may not perceive its efficiency. It is only when they assume the role of co-creator or editor that they begin to experience the full potential of AI efficiency.

The findings also suggest that cognition and understanding of AI, along with AI collaboration, mediate the relationship between mindfulness and the practical application of AI to a certain extent. Specifically, AI collaboration mediates the relationship between mindfulness and AI usage, indicating that mindfulness not only directly influences the actual use of artificial intelligence but also indirectly affects it through the two dimensions of AI literacy. First, individuals with a high level of digital mindfulness show the most significant positive impact on cognition and understanding followed by executive regulation, memory, and comprehension functions. The effect of digital mindfulness on cognition and understanding is particularly strong, as it helps deepen individuals’ understanding and knowledge of technology, thereby fostering the future development of digital applications (Liebherr et al., 2024). Second, individuals with higher levels of mindfulness are better able to reduce stress, anxiety, and fear when collaborating with intelligent technologies, thus improving their overall well-being. This, in turn, has a positive effect on their psychological well-being and further influences their intention to use AI tools by enhancing their ability to collaborate with both technologies and agents Ioannou et al. (2024).

### 5.3 The facilitating advantages of mindfulness for the application of AI technologies

Mindfulness is recognized as a psychological state of awareness that plays a vital role in helping individuals make informed decisions, reduce technological anxiety, and prevent intellectual overload. It has a positive effect on enhancing well-being during technological interactions Xu et al. (2022). First, mindfulness is crucial for reducing technological stress and emotional exhaustion during the digital and smart transitions (Azpíroz-Dorronsoro et al., 2024). “Technological Stress,” which often arises from perceived threats in the realm of artificial intelligence, is characterized by emotional reactions and resistance to technology, both of which can hinder effective use of AI. Addressing technological stress is essential, as existing research indicates that mindfulness can help to reduce this stress and alleviate “technological burnout” (Ioannou and Papazafeiropoulou, 2017). Second, “technological conflict” is another significant factor that impacts human-computer interactions. Individuals with lower mindfulness levels tend to struggle with managing the pressure induced by technological conflicts (Yang et al., 2024). Mindfulness can alleviate the subjective stress stemming from these conflicts by enhancing self-regulation and awareness, which in turn optimizes cognitive and behavioral responses (Balconi et al., 2019). Third, mindfulness is effective in controlling and alleviating “technology anxiety.” Research shows that individuals with high levels of mindfulness in the workplace experience significantly lower levels of technology-related anxiety. These individuals, with both high mindfulness and low anxiety, tend to handle conflicts more effectively (Jaiswal et al., 2019). Overall, individuals with high mindfulness levels are more likely to perceive technology as useful and select smart tools that align with their personal characteristics. As a result, they are more likely to continue using these technologies post-adoption, driven by their perception of usefulness and their ability to select technology that meets their needs (Sun et al., 2016).

Moreover, mindfulness can create subtle complexities in the relationship between mental health outcomes and emotion regulation (Curtiss et al., 2017). There is substantial empirical and theoretical evidence supporting the idea that mindfulness significantly enhances personal well-being, especially in the context of technology use. Many studies have examined this phenomenon from an individual perspective (Oeij et al., 2022). Researchers suggest that mindfulness plays a key role in alleviating psychological and physical symptoms such as anxiety and depression, and is a strong predictor of overall personal well-being (Taylor et al., 2021). Additionally, it has been proposed that “technological intrusions” can lead to abnormal behaviors, whereas IT mindfulness—defined as an employee’s focus on creatively using information technology, learning, and discovering more efficient ways to use it, which can serve as a moderating factor. Specifically, IT mindfulness helps buffer the negative effects of technology intrusion, thereby enhancing individuals’ perceptions of technological well-being (Chen Y. et al., 2022). In the “digital workplace,” mindfulness has been shown to reduce stress, overload, anxiety, and technology addiction. It also boosts employees’ technology self-confidence. Studies have demonstrated that higher levels of mindfulness not only protect and enhance employees’ personal well-being but also promote healthier digital habits (Marsh et al., 2024). Therefore, as individuals continue to develop their mindfulness traits, they become better equipped to regulate their emotions and manage technological burdens. This, in turn, helps cultivate their mental health and well-being, making them more likely to adopt and utilize AI tools in the long term (Khedhaouria and Maier, 2025).

## 6 Research conclusions and theoretical contributions

### 6.1 Conclusion

This study explores the impact of mindfulness on AI literacy and AI technology adoption in Chinese media students. Based on the analysis of survey data from 588 media students, the findings reveal that mindfulness significantly and positively predicts various aspects of AI literacy, including cognition and understanding of artificial intelligence (AI), Collaboration in AI, and Evaluation of AI in the context of media education. Within the framework of AI literacy, the cognition and understanding of AI, as well as AI collaboration, directly influence the intention to use AI tools. Furthermore, both cognition and understanding of AI and AI collaboration partially mediate the relationship between mindfulness and the intention to use AI tools. However, the self-efficacy of AI is not influenced by mindfulness. Enhancements in AI efficiency are primarily driven by practical experience, learning strategies, and technical expertise, rather than by an individual’s psychological condition. This suggests that “rational self-efficacy” is influenced by a variety of factors, including complexity and context. In comparison to other personal traits, mindfulness plays a pivotal role in improving emotional responses to technology. By reducing potential challenges such as “technology anxiety,” “numeracy stress,” and “skill burnout,” mindfulness contributes to greater technology satisfaction and well-being. Furthermore, mindfulness enhances AI literacy and facilitates more effective technology usage. In general, mindfulness can be considered an endogenous driver for the development of AI literacy and the successful integration of AI technology training, especially among students majoring in media.

### 6.2 Theoretical contributions

Based on the existing research, this study contributes to Mindfulness and Artificial Intelligence Literacy theory in several key areas.

Expanding the Application of Mindfulness Theory: Previous research has primarily applied mindfulness in fields such as psychology, health sciences, and education, where it has been used to address areas like emotion regulation, stress management, and enhancing attention. However, the intersection of mindfulness and AI literacy has not been extensively explored. This study introduces mindfulness into the framework of AI literacy, developing a theoretical framework of “Mindfulness - AI Literacy - Technology Behavior.” Through this construct, it explores potential links between personal traits and intelligence literacy, offering a novel perspective on the integration of psychology, intelligent technology, and education science.

Deepening the Multi-Dimensional Understanding of AI Literacy: In the age of artificial intelligence age, the definition of AI literacy is constantly evolving. This study refines the framework of “Cognition-Assessment-Norm-Effectiveness” within the context of mindfulness. It emphasizes the interaction between personal traits such as emotion, attention, perception, and well-being, and the cognitive and behavioral aspects of AI. Through this, the study presents a more comprehensive, balanced, and standardized framework for understanding and developing AI literacy.

Providing Insights into the AI Literacy Construction and Technology Use of Media Education: This study focuses on AI literacy construction and AI technology application of media students, a group significantly influenced by the transformative nature of artificial intelligent technologies. College students majoring in media were selected for this study due to their heightened sensitivity to information and adaptability to technology. This group also faces an urgent need to cultivate cross-media thinking and innovative capabilities, particularly in the era of intelligent media. By focusing on media majors, the research highlights how mindfulness can improve media literacy and increase the willingness to adopt AI tools, making an important contribution to educational practices in this field.

### 6.3 Practical implications

This study examines the impact of integrating mindfulness into media education on enhancing college students’ intelligence literacy and their intention to use technology. It also provides practical insights and strategies for improving AI literacy in higher education through direct and effective methods. Higher media education institutions can develop a standardized, systematic, and actionable mindfulness training program to help media students improve emotional regulation, concentration, resilience to technological stress, cognitive flexibility, and ultimately enhance their intelligence literacy and the use of intelligent tools. Specifically, universities could incorporate regular mindfulness meditation sessions into the curriculum. For example, short mindfulness exercises could be scheduled before each class or before the applications and collaborating with AI technologies to help students maintain focus and clarity in the fast-paced and complex learning environment. Moreover, mindfulness training can help students manage their emotions, reduce anxiety, and better adapt to learning and practicing new technologies. It can also boost self-confidence, happiness, and operational skills when using complex technological tools. Mindfulness has the potential to foster positive emotions, thus supporting the development of AI literacy. The application of mindfulness not only promotes mental health but also enhances adaptability, stress resistance, and innovation in the face of complex and evolving technological environments. This, in turn, strengthens students’ competitiveness and core capabilities in the artificial intelligence-driven landscape. By integrating mindfulness into media education, media education institutions can not only improve students’ intelligence literacy but also equip them with the tools to face technical challenges, “black box” risks, and algorithmic pressures with a more positive attitude, thereby helping them develop comprehensive skills and psychological resilience for future work. In addition, media education institutions can apply the principles of Information Systems Design Theory (ISDT) to design AI-assisted tools, including products, services, and user interfaces, in order to promote the sustainable integration of AI technology within media (Liu et al., 2021).

## 7 Research limitations and future prospects

Due to material resources, project funds, and time, this study still has the following research limitations and deficiencies: (1) the Comprehensiveness of Scale. This study employs a four-dimensional intelligent literacy scale to assess subjects’ autonomous AI literacy levels. Although the instrument integrates existing validated scales, the rapidly evolving nature of AI technologies may result in measurement limitations that do not fully capture respondents’ actual AI competency profiles(2) The Representative of Sample: The sample in this study is primarily concentrated in Fujian Province, China, with a relatively small sample size and a narrow research scope. Whether the sample is representative and whether the research results can be generalized still need further investigation and verification. It is recommended to expand the sample to include more regions to enhance the generalizability and external validity of the findings. (3) the Lack of Qualitative Research: This study mainly uses Structural Equation Modeling (SEM) for empirical analysis but lacks further validation of the data results. It also does not incorporate in-depth interviews or focus group qualitative data analysis, and it does not measure objective behavior, which limits the individual accuracy of the results. It is suggested that qualitative research methods be introduced or a mixed-methods approach to improve the credibility and depth of the results.

In terms of the limitations of this study, the following suggestions are proposed for future research: (1) Expanding the Sample Size: Future studies should broaden the sample size by incorporating students from universities and graduate schools across different regions, thereby enhancing the credibility, universality, and representativeness of the findings. (2) Incorporating Additional Variables: It is recommended that future research consider the inclusion of “literacy” as a key influencing factor and measurement indicator. This would allow for a more comprehensive assessment of the characteristics of students, offering a more holistic view of the variables affecting students’ use of intelligent technologies. (3) Using a Mixed Research Design: Future research could enhance the dataset by integrating multiple perspectives for cross-validation and conducting long-term longitudinal studies to explore the lasting impact of mindfulness on students’ AI literacy and behavior. Combining questionnaire surveys with controlled experiments, along with a mix of longitudinal and cross-sectional studies, will increase the validity of the conclusions and make the results more robust and generalizable. This hybrid approach will offer a clearer understanding of how students’ use of smart tools evolves over time. (4) In-depth Qualitative Research: Future studies could further enrich their findings by incorporating in-depth qualitative research methods, such as focus group discussions or interviews with media students who use AI tools. This would provide deeper insights into the nuanced experiences and perspectives of students.

## Data Availability

The raw data supporting the conclusions of this article will be made available by the authors, without undue reservation.
